# Region of interest and directional analysis of subbasal nerves in wide-area corneal nerve plexus mosaics in type 2 diabetes mellitus

**DOI:** 10.1038/s41598-020-67737-5

**Published:** 2020-07-01

**Authors:** Reza A. Badian, Tor Paaske Utheim, Neil Lagali

**Affiliations:** 1 Unit of Regenerative Medicine, Department of Medical Biochemistry, Oslo University Hospital, Oslo, Norway; 2 Department of Ophthalmology, Oslo University Hospital, Oslo, Norway; 3 Department of Ophthalmology, Sørlandet Hospital Arendal, Arendal, Norway; 4 Department of Ophthalmology, Institute for Clinical and Experimental Medicine, Linköping University, Linköping, Sweden

**Keywords:** Biological techniques, Biomarkers, Medical research

## Abstract

In vivo confocal microscopy (IVCM) imaging of the corneal subbasal nerve plexus (SBNP) is a clinical imaging modality gaining popularity for the diagnosis and follow-up of corneal neuropathy in various conditions such as diabetes mellitus. There remain, however, major limitations to the method, hindering its widespread clinical use. Finding the same exact area of the central cornea to standardize image acquisition is difficult without a reference point. Alternatively, creating wide-area mosaics of the SBNP is resource-intensive and has not yet been developed for routine clinical use. Here, we investigated whether IVCM analysis of the corneal SBNP in a predetermined, anatomically standardized region of interest (ROI) could be applied as an equivalent substitution for wide-area SBNP mosaic generation and analysis. Furthermore, we investigated nerve patterns outside the central corneal region for a possible relationship to type 2 diabetes mellitus status using a publicly available dataset. We found that corneal nerve fibre length density (CNFL) based on the ROI underestimated the mosaic-based CNFL by an average of 34% in 90% of cases (150 eyes), and did not exhibit a significant reduction with diabetes, as seen in the full SBNP. Outside the central cornea, nerve orientation differed depending on the anatomic region (left, central or right superior plexus, P < 0.001). Moreover, in long-term type 2 diabetes mellitus (≥ 10 years, 28 subjects), nerve density in the left superior sector of the SBNP was decreased (P < 0.001) while that in the central superior SBNP increased (P = 0.01) relative to 35 age-matched healthy subjects with normal glucose tolerance. These results indicate that subbasal nerve degeneration in type 2 diabetes mellitus can vary according to anatomic location, and regions with potential diagnostic value outside the central SBNP may warrant further investigation.

## Introduction

The cornea is one of the most densely innervated tissues in the human body, consisting of ciliary nerves as terminal branches of the ophthalmic branch of the trigeminal nerve^1–4^. In the anterior corneal stroma, unmyelinated nerve fiber bundles penetrate the Bowman’s layer in the central and peripheral cornea, thereafter dividing and forming a dense plexus with nerve bundles running parallel to the corneal surface, beneath the corneal basal epithelial layer. Nerve fibres that constitute the sub-basal nerve plexus (SBNP) supply the overlying corneal epithelium^4–8^. The nerves of the SBNP have multiple functions, including maintenance of corneal sensitivity to external stimuli, maintaining a healthy epithelial surface, and releasing neuropeptides and growth factors for maintaining normal corneal epithelial metabolism^9,10^. Besides these important functions, the SBNP serves as a landmark feature in the cornea, which that has been used extensively as a proxy measure of the neurotrophic status of the cornea and as an indicator of the onset and progression of systemic neurodegenerative disease^11–14^.

In vivo confocal microscopy (IVCM) is a unique, non-invasive clinical method of corneal imaging facilitating the examination of corneal layers, and can produce detailed images of the SBNP at the cellular level^15,16^. IVCM provides very high resolution, i.e. lateral resolution of 1–2 µm and axial resolution of 5–10 µm, with up to 600 × possible magnification^17^. Due to this relatively high magnification, the field of view of a single IVCM image is only 400 × 400 µm, representing only 0.2% of the corneal area. To overcome this limitation, several recent studies have proposed mosaicking individual image frames to produce wide-area depictions of the SBNP^15,18–21^. While these depictions enable more robust assessment of SBNP parameters, generating them requires considerable effort by a well-trained clinical operator and the need for separate post-processing algorithms and appropriate quality assurance measures^21^. Therefore, there may exist a trade-off between maximizing the area of the SBNP from which information is gathered and the need for a relatively simple clinical examination and image analysis procedure.

One advantage of the increased information content of wide-area images of the SBNP is the opportunity to analyze patterns in the subbasal nerve architecture across the plexus. Prior studies of the SBNP have described a characteristic whorl region or a vortex pattern where nerve fibres converge in a spiral pattern towards an area near the corneal apex, which is approximately 1–2 mm inferior and nasal to the corneal apex, located in the lower nasal quadrant^15,22^. This convergence of subbasal nerves often forms a clockwise-oriented whorl complex, where the mean nerve density in this region has been described as being significantly higher than that of the central cornea^8,15,23,24^. A study of healthy individuals suggested a marked similarity between the right and left eyes of each subject with respect to SBNP configuration and clockwise pattern in both eyes of each subject, with no mirror image symmetry between the right and left eyes and no statistical difference in the SBNP density between the eyes^25^.

Comparison of corneal subbasal nerve fiber length density (CNFL) in the central SBNP versus the whorl region in one study indicated a significantly positive correlation, with CNFL in the whorl region having a smaller coefficient of variation than that of the central cornea^26^. Another study indicated that both central and whorl regions were equally accurate for diagnosing diabetic peripheral neuropathy, with a weak significant linear relationship for CNFL-centre and CNFL-whorl versus neuropathy disability score^27^. Conversely, however, another study using wide-area mosaics showed that nerves in the whorl region were preserved and did not degenerate significantly with progression of type 2 diabetes mellitus^28^. Because the whorl region is an obvious anatomic landmark in the SBNP, less attention has been paid to nerve patterns outside the (infero-)central zone. It remains to be determined whether other SBNP regions could be sensitive to nerve pathology or degeneration, or could have potential prognostic or diagnostic value.

Here, we explore the nerve presence in the full SBNP using mosaics versus a smaller region of interest (ROI) in the central cornea that is more easily and rapidly acquired and analysed in a clinical setting. A key feature is that the ROI is referenced to a fixed location relative to the whorl centre, to improve standardization and traceability of acquired images. We aimed to deduce whether the loss of information content based on imaging a smaller area could have a diagnostic consequence for a specific disease population with type 2 diabetes mellitus. In addition, we examined the more general SBNP architecture outside the whorl and central regions to determine if the nerve density and pattern of nerves could reflect the clinical condition.

## Methods

### Study participants and dataset

The IVCM data used for the analysis of the wide-area SBNP mosaics in this study was the same raw data obtained from a previously reported cohort of subjects with type 2 diabetes mellitus, and are available openly via the Figshare platform, as previously reported^21,28^. The initial image acquisitions were performed using a Heidelberg Retinal Tomograph III with Rostock Cornea Module (HRT3-RCM), Heidelberg Engineering, Germany. The RCM has a mounted side camera, which was used to ensure proper contact between the confocal lens covered with a plastic shield/ Tomocap. The side camera aids the examiner with rough alignment to the cornea as it provides a side view of the cornea. Briefly, wide-area mosaics of the SBNP (mean area, 5.95 mm^2^) derived from 3D IVCM scanning of the plexus projected onto a two dimensional (2D) plane were obtained. In total, 163 mosaics were available, representing bilateral eye data from 81 subjects and unilateral data from one subject. The subjects were either healthy with normal glucose tolerance (NGT, 35 subjects), had impaired glucose tolerance (IGT, 8 subjects) or had type 2 diabetes mellitus (39 subjects: 28 with long-term diabetes for ≥ 10 years). In a previous study, we investigated nerve density in the entire SBNP mosaic (mCNFL) and in the whorl region (wCNFL)^28^. The wide-area mosaic images of all study subjects was available and was subjected to the analysis.

### ROI analysis

We postulated whether analysis of nerve density in the ROI could be a substitute for wide-area image analysis of the SBNP. An ROI was selected as a region in the central cornea that would be easiest to access and image reproducibly in a clinical IVCM examination. The ROI was superior to the whorl region, with its location referenced to the whorl center. As the whorl region is the most significant landmark in the SBNP, referencing the ROI to the whorl center would enable consistency and standardization in localizing the same corneal region relative to the whorl center in each examined eye. This could be relevant for comparisons of nerve parameters across individuals, but also for repeated within-subject examinations, for example in subsequent clinical follow-up visits. In this manner, we aimed to investigate the utility of the ROI for possible clinical use.

**Figure 1:**
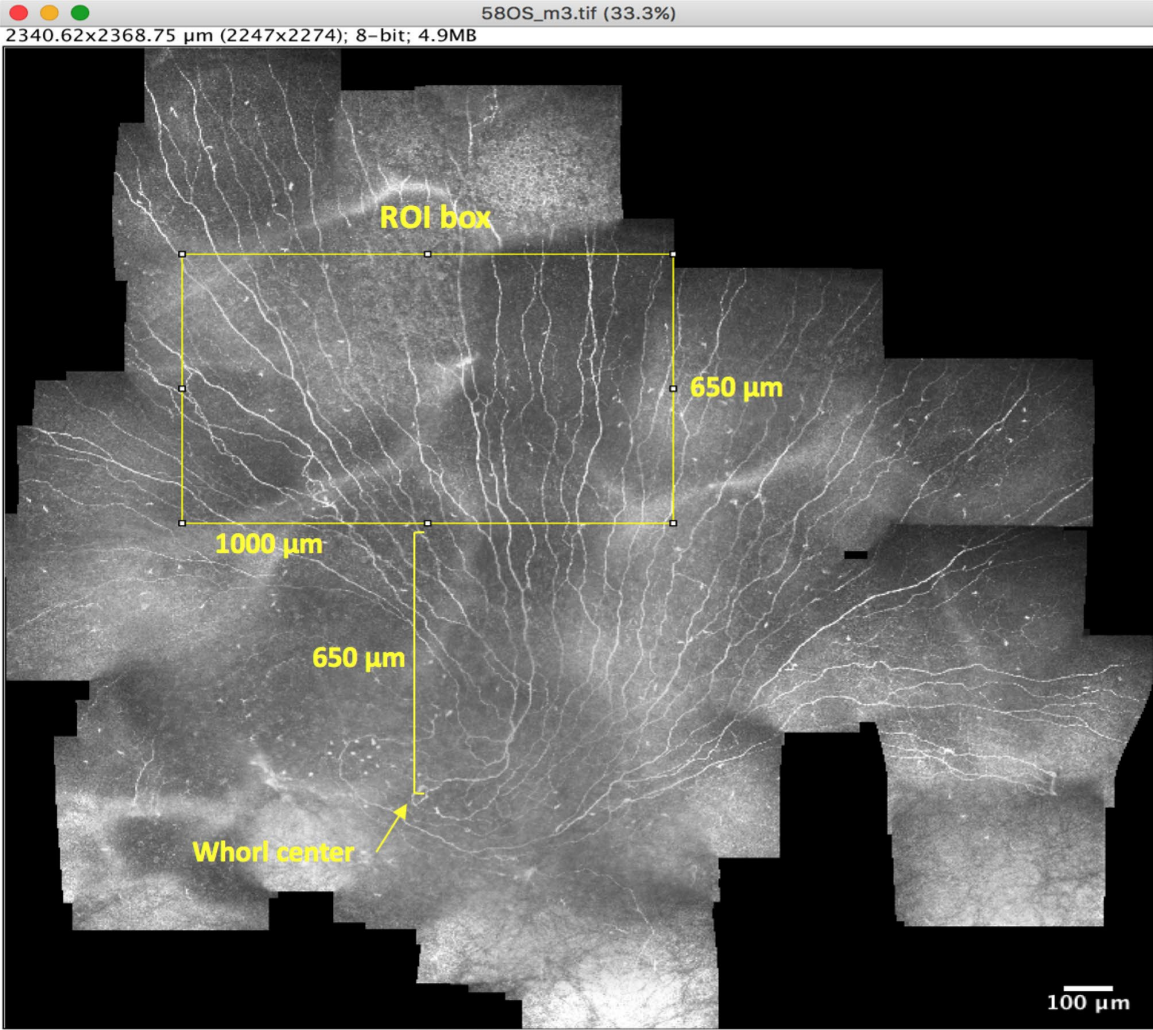
Illustration of ROI box. The ROI box (1,000 × 650 µm) is illustrated (yellow rectangle) in a mosaic as it is placed in the predefined position in relation to the whorl center (650 µm superior to the whorl center) where the center of the lower horizontal side of the ROI is positioned exactly 650 µm above the whorl center. The vertical distance between the two is marked by the yellow square bracket. The ROI was subsequently cropped from the mosaic image.

The ROI was selected by first creating a rectangular ROI box with the dimensions 1,000 µm (width) and 650 µm (height) using ImageJ. This ROI represents an area of four times the standard IVCM image frame size and was deemed suitably large to yield a broader view than a single frame while being small enough to be feasible to obtain in most eyes within a clinical setting. The coordinates of the whorl center were then identified from the mosaic image, and the ROI box was placed superior to the whorl center such that the centre of the lower horizontal side of the rectangle was positioned directly 650 µm superior to the whorl center (Fig. 1). With the ROI rectangular box positioned above the whorl centre, the ROI area could be cropped from the wide-area mosaic image. Two experienced observers conducted the ROI analysis; the first observer analysed 100% of ROI images and the second observer analysed 25% of images in each of the four subgroups. The cropped ROI region was then subjected to quantitative analyses of the following parameters.

1. Number of main nerves: main nerves were defined as nerves that traversed the height of the ROI area from top to bottom.
2. Main nerve fibre length density: main nerves in the ROI were traced using the NeuronJ^29^ plugin for ImageJ. Subsequently, the total length of the main nerves in the ROI was determined and divided by the ROI area.
3. Total nerve fibre length density: all nerve fibres in the ROI, both the main nerves and secondary branches were identified and traced. The total length of all nerves in the ROI was divided by the ROI area, and considered the CNFL in the ROI.
4. Secondary nerve fibre length density: secondary nerve fibres were defined as all the nerve fibres other than the main nerves. The combined length of all secondary nerve fibres in the ROI was divided by the ROI area.
5. Nerve branch density: this refers to the sum of the number of points within the ROI where nerves bifurcate into branches, divided by the ROI area.
6. Inter-nerve distance: this is the average distance between the main nerves in the ROI area. First, a horizontal line bisecting the short sides of the ROI box area was drawn. Then, the distance between each main nerve and the adjacent main nerve was measured in ImageJ using the line tool. The mean value of all such distances in the ROI was then determined.

**Figure 2:**
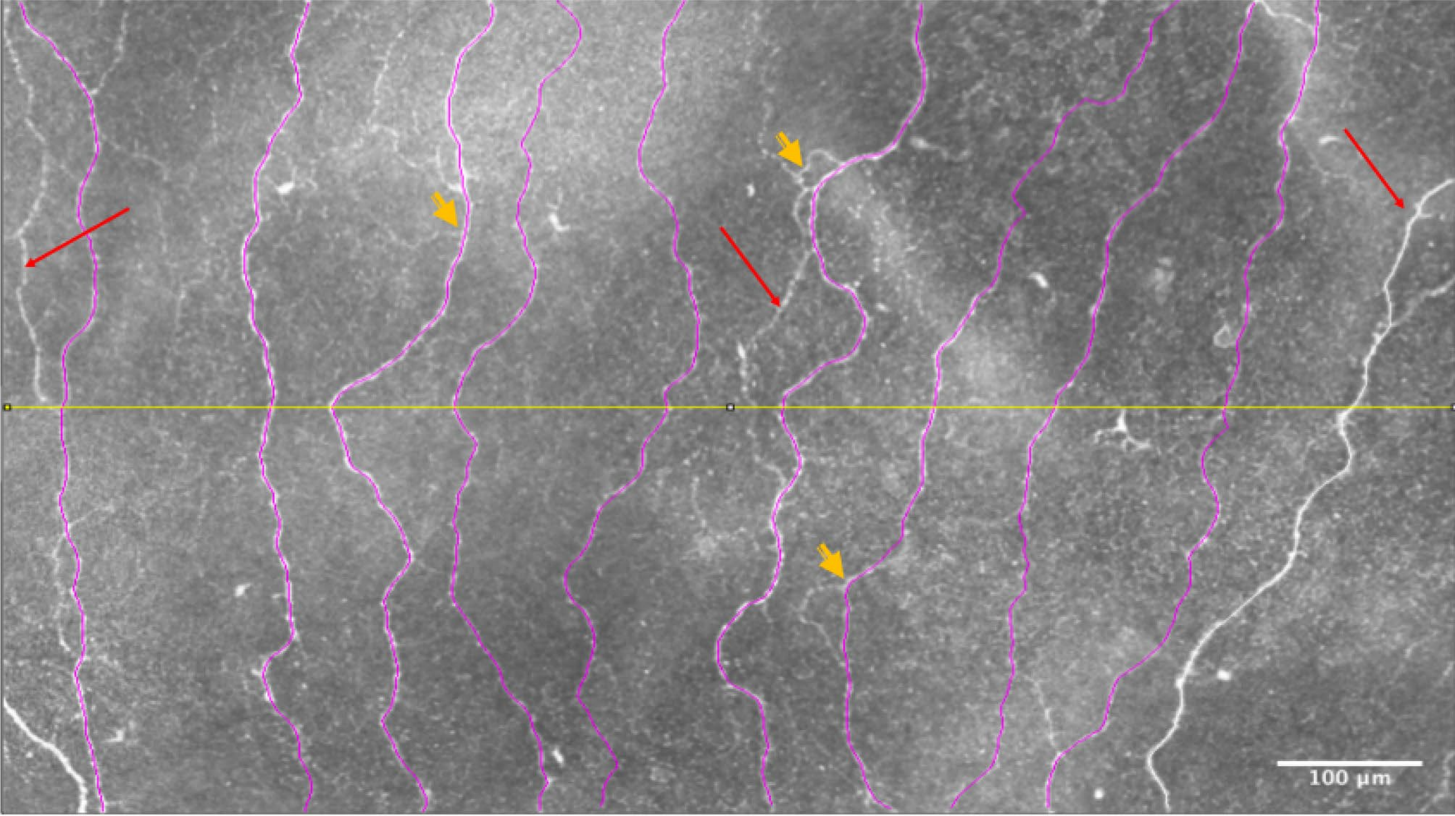
ROI image illustrating different parameters. A region of interest (ROI) cropped from an original wide-area mosaic image is shown here. The main nerves that transverse from the top to the bottom of the image are traced (marked in purple). Red arrows indicate the secondary nerves. Short yellow arrows indicate nerve bifurcations (branches). White double-headed arrows indicate the inter-nerve distance (IND). The mid-horizontal line (in yellow) divides the image at the height of 325 µm. One nerve to the right of the image (in white) was not included among main nerves because it did not satisfy the condition of traversing from the top to the bottom of the ROI image. The ROI image size is 1,000 µm (width) × 650 µm (height).

Figure 2 illustrates these definitions graphically. The above parameter values were determined for the right and left eyes of each subject, with the observer masked to the subject’s identity. Subsequently, the parameters across both eyes of the same subject were averaged. In an earlier study^28^, we measured the values for total nerve fiber length density in the entire (non-cropped) mosaic (mCNFL) and similarly averaged them across both eyes of a given subject. These values served as a comparison to the corresponding nerve density measures in the ROI.

### Nerve orientation analysis

As the patterns of innervation across the SBNP were not visible in the ROI, we analysed patterns in the wider SBNP by considering the hemi-plane of the wide-area mosaic images located superior to the whorl center. In practice, this region of the SBNP had the best coverage and was most consistently imaged within the dataset. Orientational status of nerves was assessed by semi-quantitatively determining the predominant directionality of the main nerve fibres in three sectors of the superior hemi-plane. These sectors were delineated by considering the whorl’s hemi-plane axis as extending from 0° to 180° in a clockwise manner for both right and left eyes as indicated by Misra et al.^25^. The sectors were categorized as follows:

Left side of SBNP: 0°–60°, temporal side in right eye or nasal side in left eye.

Central SBNP: 60°–120°.

Right side of SBNP: 120°–180°, temporal side of left eye or nasal side of right eye.

The specification of all sectors with respect to their respective anatomical location and coordinates in both eye is shown in Table 1.

**Table 1:** The specification of sectors in both eyes with respect to their equivalent anatomical positions and respective sector coordinates.

Right (OD) or left eye (OS)	Location of sectors in the superior hemiplane	Sector coordinate (°)	Anatomical specification of sectors
Right eye (OD)	Left side of SBNP	0**°**–60**°**	Superior temporal
Right eye (OD)	Central SBNP	60**°**–120**°**	Cranial
Right eye (OD)	Right side of SBNP	120**°**–180**°**	Superior nasal
Left eye (OS)	Left side of SBNP	0**°**–60**°**	Superior nasal
Left eye (OS)	Central SBNP	60**°**–120**°**	Cranial
Left eye (OS)	Right side of SBNP	120**°**–180**°**	Superior temporal

The angles (0**°**–180**°**) are measured clockwise in each hemiplane.

*OD* Oculus Dexter, *OS* Oculus Sinister.

**Figure 3:**
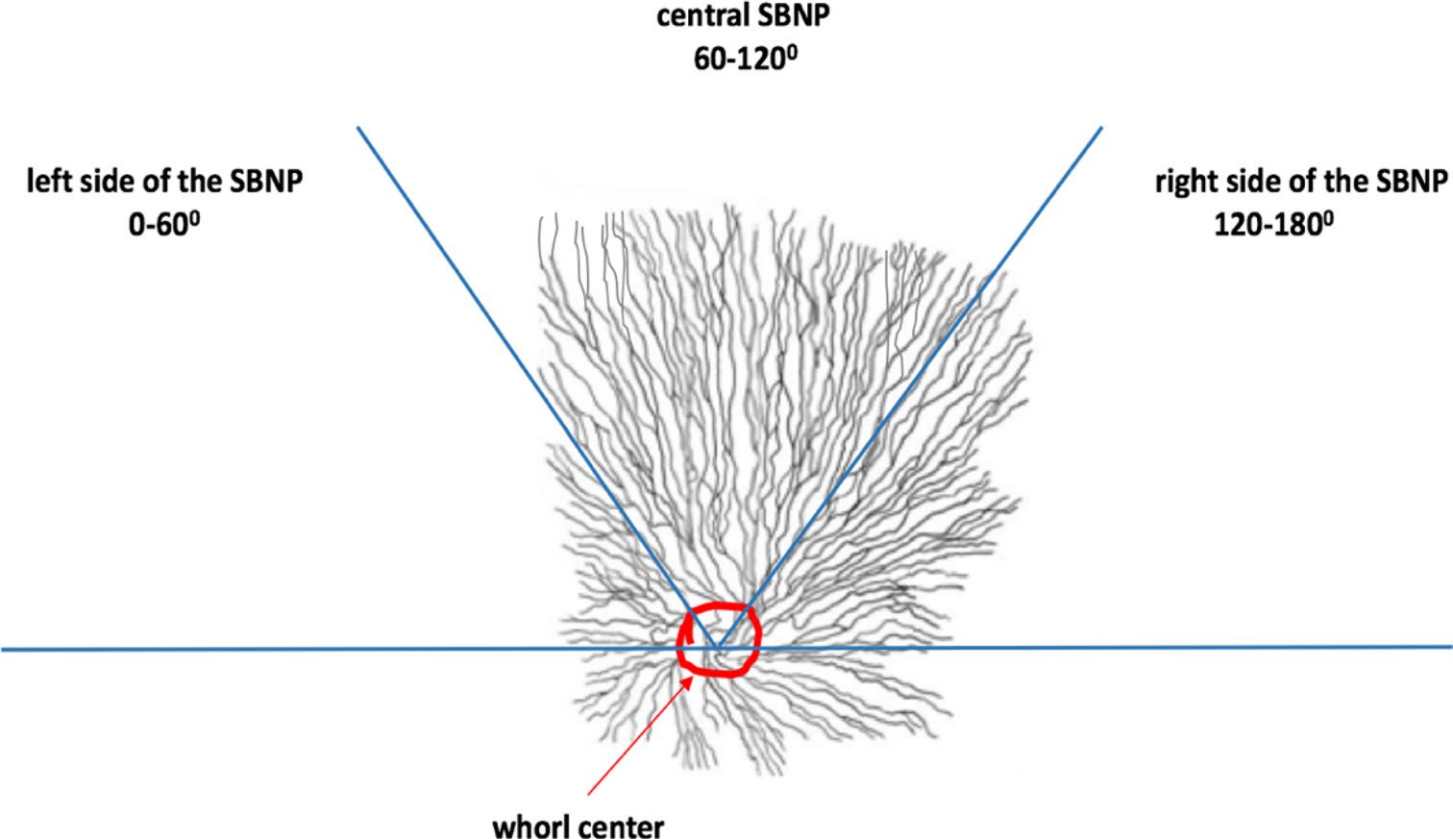
Schematic illustrating the division of the SBNP into a superior hemi-plane defined by a horizontal line intersecting the whorl center (circled in red). Within the superior plexus, three sectors were defined, each comprising a 60° arc: left, central and right sides of the SBNP. Nerve orientation and density within these sectors were analysed semi-quantitatively.

Within each sector (left, central or right), the predominant orientation of the main nerves was graded as northwest-to-southeast (NW–SE), north-to-south (N–S) or northeast-to-southwest (NE–SW), which were then coded as 1, 2 and 3, respectively (Fig. 3). The density of main nerve fibres in each sector was semi-quantitatively graded based on the results of a preliminary analysis, which indicated that the mean number of main nerves in a given sector was approximately 6 ± 2. The density in each sector was therefore graded as low (number of main nerves < 4), medium (between 4 and 8 main nerves) or high (> 8 main nerves).

### Statistical and power analysis

Statistical comparisons were performed using one parameter value per subject (average value of both eyes), except where comparing nerve density between different methods of measurement (whole mosaic versus ROI). The CNFL and mCNFL in the ROI were compared using the Mann–Whitney test, and ROI-based nerve parameters between subject groups of NGT and long-term diabetes were compared using independent *t* tests. Nerve density and directionality were measured by semi-quantitative grading were compared across left-side, central and right-side regions of the superior SBNP and across NGT and long-term diabetes groups using one-way repeated measures analysis of variance (ANOVA); pairwise differences were isolated using the Tukey post hoc test.

Statistical power calculations were performed using a standard formula for power and sample size^30^. Sample size was the sum of study subjects in the NGT and long-term diabetes groups, with critical significance level set to α = 0.05 and false negative rate set to β = 0.20 (80% power).

## Results

### Full mosaic versus ROI region comparison

**Figure 4:**
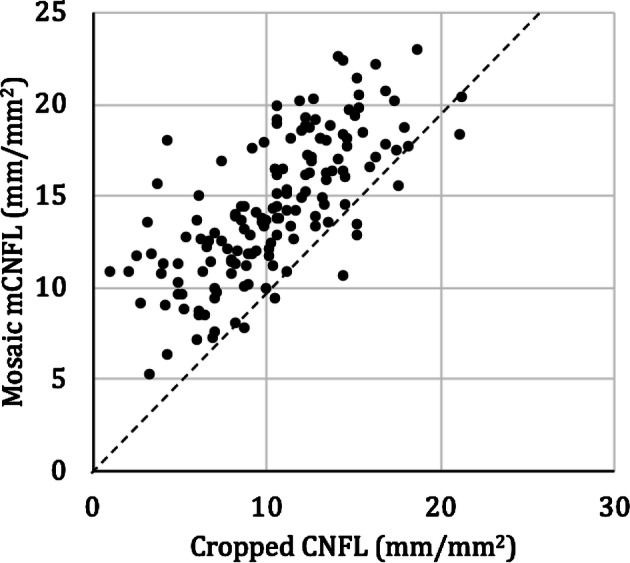
Comparison of mosaic mCNFL vs ROI CNFL (cropped region). The dashed line indicates equal CNFL values. Each data point represents a single eye. Data points above the dashed line indicate where the CNFL was underestimated by the ROI (cropped region).

**Figure 5:**
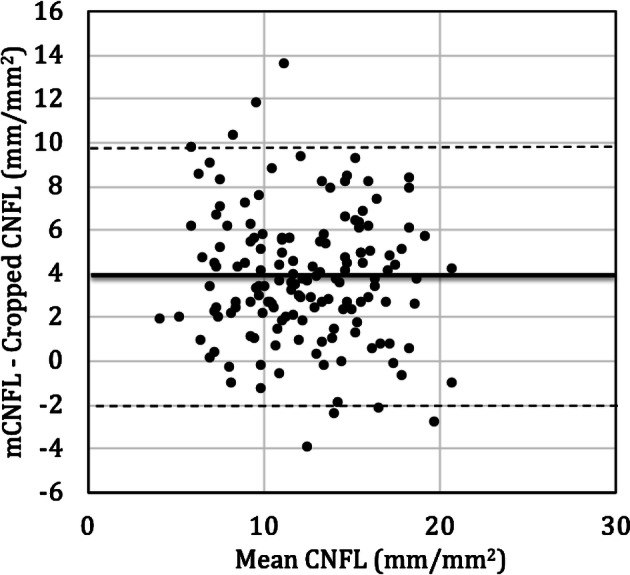
Bland–Altman analysis illustrating the difference in CNFL estimates based on cropped ROI or full mosaic (mCNFL). The difference in CNFL between the approaches was plotted versus the mean of both methods. The thick black line indicates the mean difference; while the dashed lines indicate the 95% lower and upper limits of agreement, respectively, with the values indicated in the main text.

**Figure 6:**
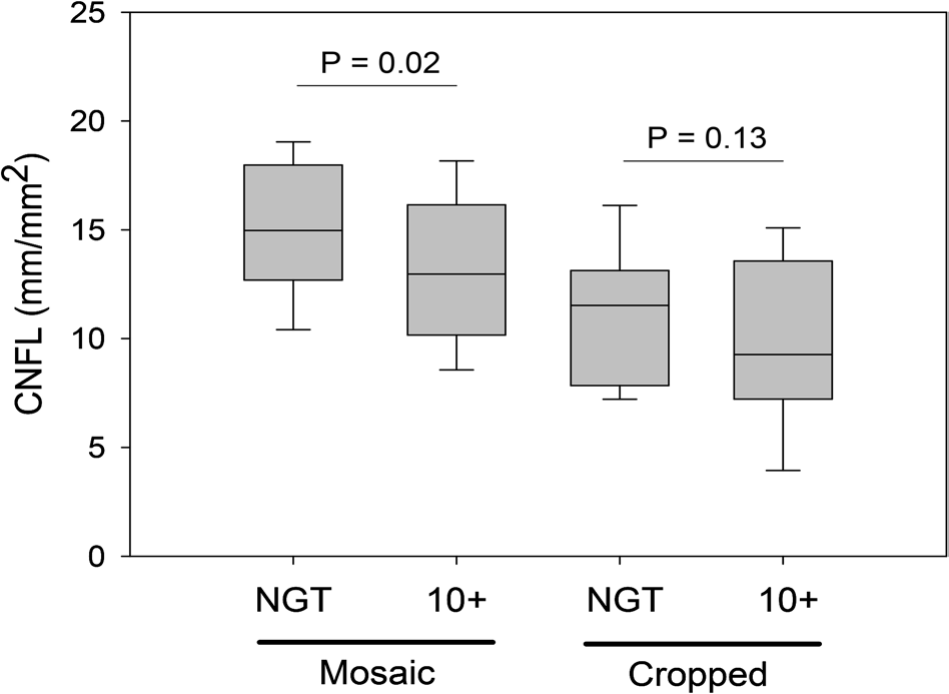
Box plot comparing mosaic vs cropped images of a region of interest (ROI) in the SBNP in NGT and long-term diabetes (> 10 years) groups in terms of subbasal nerve density (CNFL). Using the entire mosaic, a significant decline in CNFL was detected, which did not persist when only the cropped ROI was considered.

From the mosaic dataset of the entire cohort, it was possible to produce 150 cropped images representing a standardized ROI with the same size and location relative to the whorl center. These represented data from 150 individual eyes. Nerve fiber density in mosaics (mCNFL) as previously reported^21,28^ was then compared to the CNFL in the ROI (Fig. 4). In 90% of cases, the ROI-based CNFL underestimated the mCNFL. Bland–Altman^31^ analysis (Fig. 5) revealed that the ROI-based CNFL underestimated the mCNFL by a mean of 3.73 ± 2.97 mm/mm^2^, which corresponds to a 34% underestimation of nerve density on average. The 95% limits of agreement for the amount of underestimation were − 2.09 to 9.56 mm/mm^2^. Considering the mean CNFL from both eyes of the same subject, the median ROI-based CNFL across all subjects represented a significant underestimation for the mCNFL (14.3 mm/mm^2^, P < 0.001). Based on our prior analysis^28^, the long-term diabetes group of 28 subjects had a significant reduction in mCNFL relative to the NGT group of 35 subjects (P = 0.02, 12.7 ± 4.2 vs 15.3 ± 3.2 mm/mm^2^; mean difference, 2.4 mm/mm^2^). For the ROI-based CNFL, however, the difference between groups was reduced by 30%, resulting in a non-significant difference of CNFL between the long-term diabetes and NGT groups (P = 0.10, 8.9 ± 4.4 vs 10.7 ± 3.6 mm/mm^2^; mean difference, 1.7 mm/mm^2^). This result is illustrated graphically by the box plot in Fig. 6.

### Nerve parameters in the ROI

Bland–Altman analysis revealed good general agreement of semi-automated nerve parameter quantification between observers. The number of main nerves per ROI differed by a mean of 0.76 nerves between observers with a 95% limit of agreement (LOA) of ± 4.3 nerves. Main nerve density differed by 0.74 mm/mm^2^ with a 95% LOA of ± 4.6 mm/mm^2^ while total nerve density (CNFL) differed by a mean of 1.47 mm/mm^2^ with a 95% LOA of ± 6.8 mm/mm^2^.

Focusing on the ROI, we quantified various nerve parameters in this region and compared them between the NGT and long-term diabetes groups, with values per subject representing the averaged values across both eyes of a given subject. The results, including the difference in means along with the required minimum difference for 80% power, are stated in Table 2. In all cases, the long-term diabetes group tended towards a pathological level of the parameter relative to NGT; however, none of the parameter differences reached the level required to detect a difference at the 80% power level.

**Table 2:** ROI parameter comparison between NGT and long-term diabetes groups.

Parameter	Healthy (NGT)	Long-term diabetes	Difference of means	P value	Minimum diff. for 80% power
Number of subjects	33	27			
Number of main nerves	6.15 ± 1.77	5.28 ± 1.90	0.87	0.07	1.32
Main nerve density (mm/mm^2^)	6.54 ± 1.91	5.88 ± 2.12	0.65	0.22	1.45
Secondary nerve density (mm/mm^2^)	11.20 ± 3.21	9.63 ± 3.92	1.56	0.10	2.58
Total nerve density (mm/mm^2^)	10.67 ± 3.58	8.94 ± 4.40	1.73	0.10	2.89
Nerve branch density (no./mm^2^)	99.0 ± 42.7	85.2 ± 51.7	13.80	0.26	34.15
Inter-nerve distance (µm)	126.4 ± 55.4	149.6 ± 70.6	− 23.2	0.11	− 45.6

P-value of independent t-test is indicated, along with minimum difference of means required to detect significance with 80% power.

*diff.* difference.

### Nerve orientation and density variations in the SBNP

**Figure 7:**
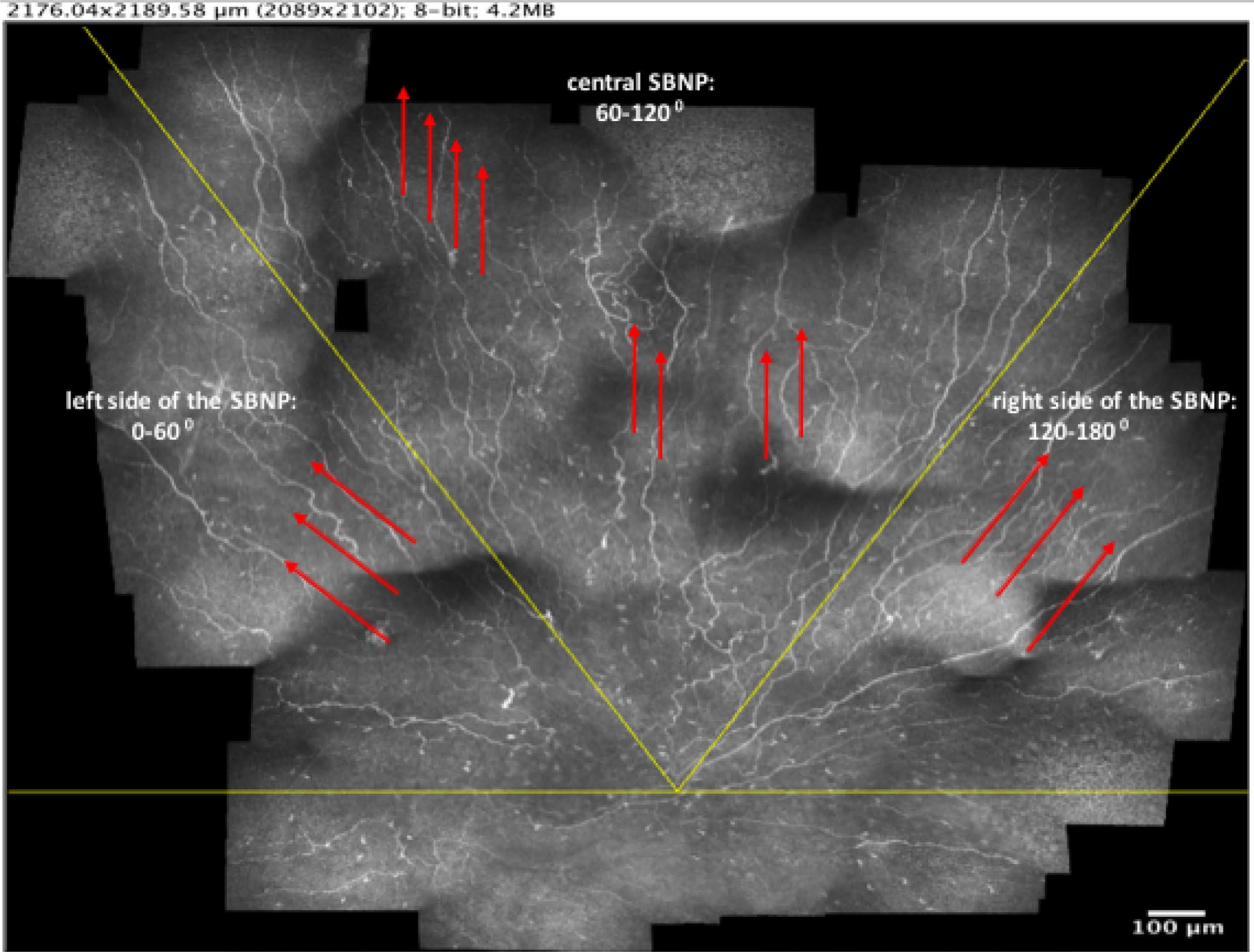
Illustration of sectors for orientation analysis in a right eye of a subject with > 10 years diabetes mellitus (DM 10 + years). The horizontal line (in yellow) through the whorl centre indicates the superior hemi-plane of the SBNP. The red arrows indicate the predominant direction of the nerves within each sector, independent of disease status.

Upon dividing the superior hemi-plane of the SBNP into three distinct regions, data from both eyes of a subject were averaged. The median orientation of nerves in the left, central and right sides of the SBNP were as follows (pictured in Fig. 7):

Left side of SBNP: NW–SE.

Central SBNP: N–S.

Right side of SBNP: NE–SW.

Nerve directionality differed significantly across all three regions (P < 0.001), while the above orientation pattern of nerves within the three regions was maintained regardless of group (NGT or long-term diabetes). Nerve density also differed across the SBNP regions (P < 0.001), being highest in the central SBNP (median density, grade 2.5) relative to left and right sides of the SBNP (median density for both, grade 2.0). Contrary to the nerve directionality, which did not change with disease status, in long-term diabetes the left side of the SBNP had decreased nerve density (median grade, 1.5) relative to the NGT group (median grade, 2.0, P < 0.001). In the central SBNP, nerve density increased in long-term diabetes (median grade, 2.5) relative to NGT (median grade, 2.0, P = 0.01). No difference in nerve density, however, was detected in the right side of the SBNP, with both groups maintaining a median nerve density grade of 2.0 (P = 0.35).

## Discussion

Here, we used as a starting point a robust open access dataset of wide-area IVCM mosaics of the SBNP in healthy subjects and subjects with type 2 diabetes mellitus^21^. For our first analysis, we chose an ROI with a size four times larger than a single IVCM image. This provided a larger area than a single image while still being a small enough area to be feasible to quickly obtain data in a clinical setting. The larger the area of the ROI, the more technically difficult it would be to consistently obtain full data for the region during the image acquisition process in all subjects. Within the available dataset used here, a larger ROI would not have been represented in all mosaics, as black pixel areas (no data) would have been present in some mosaics. This is one limitation of the original dataset, that precludes a comprehensive analysis of various sizes of the ROI to determine an optimum size and/or location.

**Figure 8:**
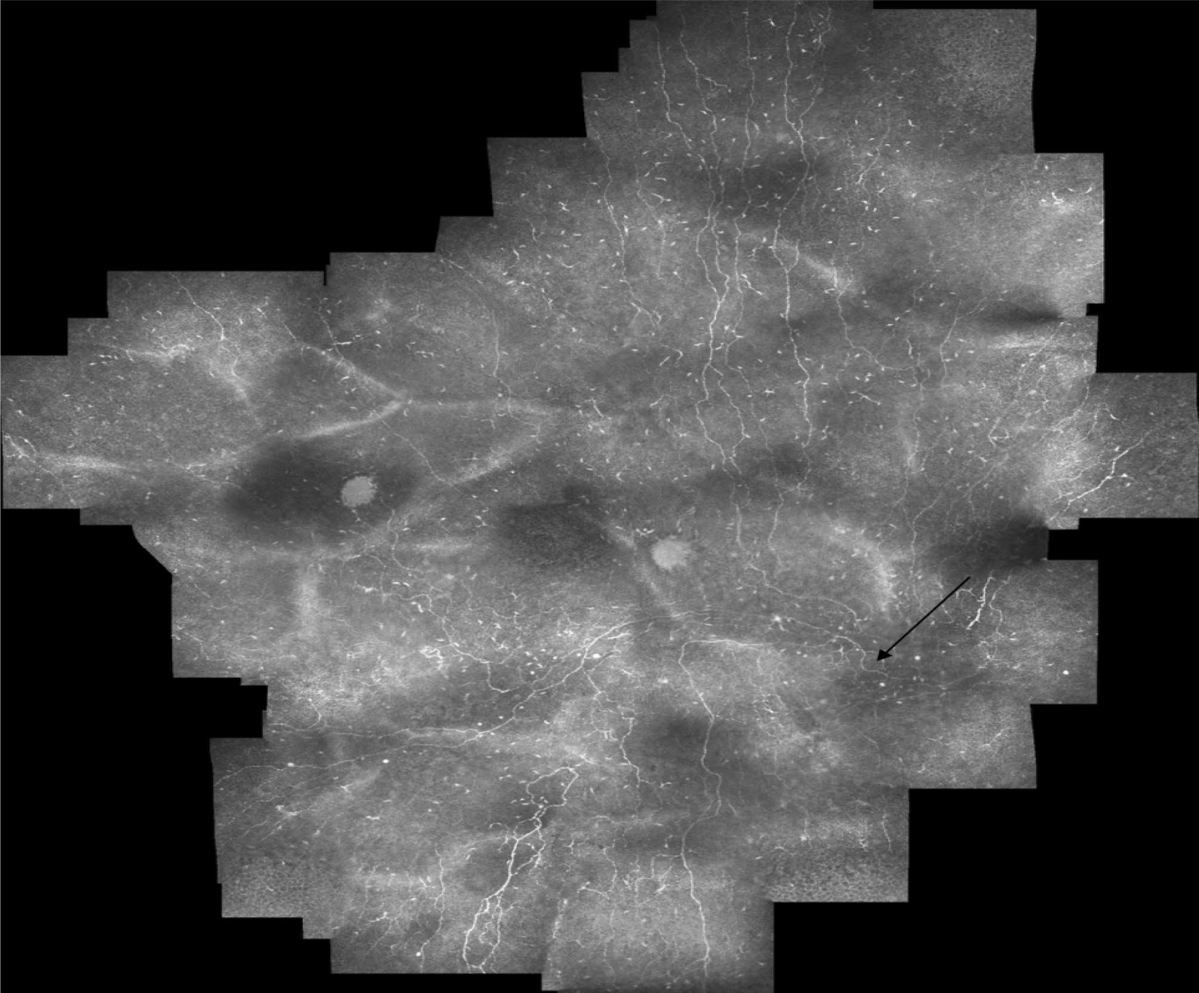
Mosaic image from subject with diabetes mellitus with duration of diabetes > 20 years and reduced nerve density (nerve density: 8.7 mm/mm^2^), where the whorl-center could be identified (black arrow).

Our first aim was to determine whether the entire mosaicking acquisition and reconstruction procedure was necessary for detecting the nerve degeneration seen in type 2 diabetes mellitus. Notably, the vast majority of studies of IVCM analysis of the SBNP use multiple single-image frames taken from the central cornea to determine an average CNFL, without reconstructing the plexus^11^. As such methods are easier to implement in a clinical setting rather than full mosaic generation, we hypothesized that an improvement upon the single-frame IVCM approach, namely using a fixed-size ROI with a standardized location in the SBNP (referenced to the whorl center), would provide a more reproducible alternative to subjective single-frame selection. The whorl-center could also be identified in mosaic images where the subbasal nerve density was reduced either in subjects with diabetes mellitus or in non-diabetic subjects (Fig. 8).

The ROI analysis results, however, revealed that CNFL based on the ROI underestimated the mCNFL by a significant amount (34%) in a significant proportion of eyes (90%). This may be the result of the ROI excluding the whorl region known to have the greatest nerve density in the SBNP^8,15,23,24^. Further investigation revealed that an ROI-based analysis would not yield significant power for detecting disease-dependent nerve degeneration (or alternatively would require larger group sizes), as nerves in the ROI representing the central SBNP varied to a lesser degree with type 2 diabetes mellitus than nerves across the entire SBNP mosaicked region. Power analysis indicated that a 67% greater difference in the ROI CNFL between groups would have been required to detect significance. Similarly, while other ROI parameter means exhibited a trend towards nerve degeneration in long-term diabetes (Table 2), no parameter difference was sufficient for detecting a difference at an 80% power level. This result indicates that a small, standardized central region of the SBNP with an area corresponding to four IVCM frames has insufficient diagnostic utility. This suggests that wide-area mosaics and mCNFL provide significant additional information relevant to the disease not available in the ROI. We therefore sought to examine nerves in the wider SBNP outside the ROI.

Interestingly, nerves in the hemi-plane superior to the whorl center had a similar pattern across the healthy and long-term diabetes groups. Nerves directly superior to the whorl center and those within the central SBNP in a 60° zone were oriented vertically in a N–S direction regardless of the presence of diabetes. Similarly, nerves on the left side of the SBNP had a negative sloped NW–SE orientation while that on the right side of the SBNP had a positive-sloped NE–SW orientation. This was the predominant pattern of subbasal nerves in subjects of the present cohort. It remains to be seen whether this pattern is similarly maintained in other cohorts, but future studies presenting wide-area mosaics of the SBNP could shed light on this question.

Despite the consistency of the general subbasal nerve orientations, however, nerve density varied with location in the SBNP. The largest nerve density decline with diabetes occurred in the left side of the SBNP (OD temporal/OS nasal region superior to the whorl), whereas nerves in the central SBNP region superior to the whorl were denser in the diabetes group. This finding indicates that nerve location within the SBNP could be an important consideration when assessing the SBNP in disease. Randomly selecting single or multiple IVCM images from an area believed to be the central cornea may yield significant differences across groups, as reported in numerous studies and noted in systematic reviews^11,32,33^; however, most studies also report substantial standard deviations for subbasal nerve parameters^27,28,34–39^. Focusing on a specific region of the SBNP that is more sensitive to nerve degeneration may therefore have the potential to reduce the variability of CNFL and thereby provide a potentially more useful diagnostic or prognostic indicator of disease.

Our results presented here require further validation in different disease cohorts. Producing wide-area mosaics of the SBNP in a clinical setting may be more labour-intensive and time-consuming than standard IVCM examinations, but such mosaics are essential for determining changes in subbasal nerves in a robust and reproducible manner. Through the use of optimized algorithms, the mosaics can be produced in a matter of minutes. For instance, we previously reported that a mosaic representing a 6 mm^2^ area of the central cornea could be produced in 7 min^21^. The equivalent time for producing the smaller ROI in this study would be approximately 45 s. A limitation of the present study, however, was the cross-sectional nature of the examinations. Future studies should additionally aim to examine the same eyes longitudinally, ideally over a longer time course, to provide stronger evidence for the changes in subbasal nerve architecture and density in relation to disease. For instance, further studies are needed to confirm our findings that subbasal nerve density increases in the central SBNP region superior to the whorl, and decreases in the left side of the SBNP in subjects with long-term diabetes relative to those with NGT. Although the SBNP has been shown to be a dynamic structure^40^, the reasons for this particular pattern of change in diabetes remain unclear.

Using fixed, stained samples, He et al. showed a significant decrease in the central subbasal nerve density in donated cadaver corneas from eight subjects with diabetes relative to six healthy donors^41^. Utsunomiya et al. conducted a comparative IVCM study of diabetic (47 subjects) versus non-diabetic groups (21 subjects), and using five single IVCM frames from the central region (non-standardized); they reported significantly reduced CNFL in diabetes^26^. Moreover, in diabetic relative to healthy corneas, both in vivo and ex vivo studies of corneal SBNP have found a reduced number of branches and fewer connections between nerve branches in the SBNP relative to the normal cornea, but image sampling was not standardized in those studies^26,35,41–43^. The lower values of nerve branching detected in the present study, however, are insufficiently robust for confirming this result.

In summary, the present study indicates that there are significant differences in the density and orientation of subbasal nerves depending on the location in the cornea where density is analysed. Standardizing the location of SBNP analysis to a whorl-referenced central corneal region four times the size of a single IVCM image frame did not improve diagnostic utility, suggesting that larger regions of the SBNP are desirable. In particular, the central nerve density, which often is used by many investigators, tended to underestimate the nerve density in the overall SBNP in the present cohort. Consequently, the central corneal region may not be representative of the entire SBNP, and may require augmentation with non-central regions for improved diagnostic value. Although in this study the central ROI did not yield discriminative power between the subject groups, it is possible that some combination of central, whorl, and peripheral regions (for example, adding, subtracting or referencing density values between anatomic regions) could result in improved sensitivity and specificity. The dataset used in this study is openly available^21^, thereby facilitating further studies.
